# Risk Factors for Elevated D-Dimer Levels in Patients with Gastrointestinal Tumors Treated with Endoscopic Submucosal Dissection

**DOI:** 10.3390/jcm12165229

**Published:** 2023-08-11

**Authors:** Shogo Komatsuda, Shinya Kodashima, Ken Ikusaka, Naoaki Aoki, Yuki Shimizu, Minoru Oda, Fumito Harada, Taku Honda, Shingo Komazaki, Miyoko Sakurai, Daisuke Yanagisawa, Kyohei Maruyama, Hitoshi Aoyagi, Akari Isono, Ryo Miura, Koichiro Abe, Toshihiko Arizumi, Yoshinari Asaoka, Takatsugu Yamamoto, Atsushi Tanaka

**Affiliations:** 1 Department of Medicine, Teikyo University School of Medicine, Tokyo 173-8605, Japan; 2 Department of Gastroenterology, Seibu General Hospital, Saitama 338-0824, Japan

**Keywords:** D-dimer, deep-vein thrombosis, endoscopic submucosal dissection, gastrointestinal tumor, sedative

## Abstract

Endoscopic submucosal dissection (ESD) is almost always performed with a sedative because of the longer procedure times involved. The risk of post-ESD deep vein thrombosis (DVT) has been reported as relatively high, and D-dimer levels are sometimes elevated after ESD. This retrospective study evaluated factors affecting changes in D-dimer levels from before to after ESD to identify causes of elevated D-dimer levels after ESD. This retrospective analysis included 117 patients with gastrointestinal tumors resected using ESD. After excluding eight patients with pre-ESD levels of D-dimer >1.5 μg/mL, factors correlating with changes in D-dimer from before to after ESD were analyzed using logistic regression analysis in 109 patients. Sedation was accomplished primarily using midazolam, but, because the sedative effect of midazolam shows marked inter-individual variability, a “corrected midazolam dose” was determined by dividing the total midazolam dose by the initial dose to correct for inter-individual differences in the sedative effect of midazolam. This value was used as one potential explanatory variable in the subgroup analysis of the 103 patients who received midazolam. In the subgroup analysis using the corrected midazolam dose as an explanatory variable, only the corrected midazolam dose correlated with a change in D-dimer ≥1.0 μg/mL in multivariate analysis (odds ratio (OR) = 1.5, 95% confidence interval (CI) 0.43–0.95; *p* = 0.030). The corrected midazolam dose correlated with increases in post-ESD D-dimer levels. This potential relationship indicates that patients undergoing ESD and requiring extended sedation may be at increased risk of DVT.

## 1. Introduction

Venous thromboembolism (VTE) refers collectively to pulmonary thromboembolism (PTE) and deep vein thrombosis (DVT) [1]. PTE can result in shock or sudden death [2], and 90% of the thrombi involved in PTE originate in the veins of the lower legs or pelvis [1]. DVT must thus be properly prevented and treated to protect the patient from such outcomes. As DVT often occurs following surgery or during hospitalization for a medical condition, prevention of DVT is essential in hospital settings. Current guidelines recommend a preventative strategy tailored to the risks involved, such as the type of procedure, the age of the patient, and the presence of cancer [1].

In recent years, endoscopic submucosal dissection (ESD) has become a widely used option for removing gastrointestinal tumors that diagnostic imaging indicates have a very low likelihood of being metastatic [3]. ESD produces fewer early complications and requires a shorter hospital stay than does surgery, providing a safer option, particularly for elderly patients [4,5,6]. ESD, however, presents some problems, including the high degree of procedural difficulty [7,8] and potentially longer procedure times [7,9,10]. Current guidelines recommend the use of sedative in endoscopic procedures to increase safety and reduce patient suffering [11]. Because of the typically longer procedure times, ESD is almost universally performed with a sedative, often requiring moderate or greater sedation.

Kusunoki et al. [12] estimated the risk of post-ESD DVT as relatively high, at 10%. That study noted that D-dimer levels were elevated in patients with post-ESD DVT, but did not elaborate on specific risk factors for DVT. As a result, prophylaxis for DVT is not generally provided with endoscopic procedures.

D-dimer is a soluble fibrin/fibrinogen degradation product produced following the degradation of stable fibrin and is an end degradation product of fibrin. D-dimer is thus indicative of elevated secondary fibrinolysis and is often considered in the diagnosis of disseminated intravascular coagulation (DIC) and VTE, including DVT [13]. D-dimer levels are also elevated in inflammation, infections, cancer, hepatic cirrhosis, pregnant women, and older individuals [1]. Although lacking diagnostic specificity for VTE, D-dimer is widely used to exclude DIC and VTE in clinical settings because normal levels indicate a lack of thrombi [14].

Nakamura et al. also reported that the D-dimer levels were sometimes elevated after ESD, especially for patients with upper gastrointestinal lesions and underlying chronic kidney disease [15]. Although they did not refer to the causes of these elevated D-dimer levels, the elevation in D-dimer levels just after ESD is likely attributable to either thrombi or inflammation, considering the mechanisms involved in the production of D-dimer.

The aim of this retrospective study was to evaluate factors affecting changes in D-dimer levels from before to after ESD to identify the cause of elevated D-dimer levels after ESD and to clarify the relationship between post-ESD elevations in D-dimer levels and post-ESD DVT.

## 2. Patients and Methods

### 2.1. Patients

Our study included patients admitted to Teikyo University Hospital (Tokyo, Japan) between November 2019 and October 2020 for ESD for gastrointestinal tumor. D-dimer levels the day before and the day after ESD were retrospectively analyzed to identity the causes of elevations in D-dimer levels after ESD. In evaluating changes to D-dimer levels between before and after ESD, patients in whom pre-ESD levels of D-dimer were >1.5 μg/mL were excluded [16] because of their possibility of DVT, and factors that correlated with a change in D-dimer levels ≥1.0 μg/mL were evaluated. D-dimer levels (Sysmex Corporation, Kobe, Japan) were determined in the routine manner in the clinical laboratory of our hospital.

### 2.2. ESD Technique

All ESD procedures were performed by or under the guidance of an endoscopist with at least 5 years of ESD experience, with no provision of DVT prophylaxis. All upper gastrointestinal (esophageal and gastric) ESD procedures were performed with the patient restrained in the left lateral position. For colonic ESD, the patient position best suited to the lesion site and maintaining scope stability was selected. Sedatives and analgesics were administered as described below while the level of consciousness, respiration, and hemodynamics of the patient were monitored. Following resection, hemostatic forceps were used for coagulation of blood vessels on the ESD ulcer.

### 2.3. Sedative and Analgesic Regimens

At our hospital, sedation was accomplished primarily using midazolam. After 0.5–2 mg of midazolam was intravenously injected, additional amounts of 0.5–1 mg of midazolam were injected as necessary while the level of sedation was monitored. The endoscopist began ESD once sedation sufficient to begin the procedure had been achieved, confirming whether patients could respond purposefully to verbal commands. The dose of midazolam used up to this point was taken to be the initial dose. Amounts of 0.5–1 mg of midazolam were injected during the procedure referring to the initial dose of midazolam when the endoscopist determined that additional sedation was necessary while the level of sedation was monitored. The sedative effect of midazolam generally shows marked inter-individual variability [16,17,18,19,20,21], so a “corrected midazolam dose” is determined by dividing the total midazolam dose by the initial dose to correct for inter-individual differences in the sedative effect of midazolam. This value was used as a potential evaluation factor in the subgroup analysis of midazolam users in the present study. When additional midazolam was insufficient to maintain sedation, haloperidol was administered in addition to midazolam.

The endoscopist decided whether to use pentazocine hydrochloride or pethidine hydrochloride for analgesia. The initial intravenous dose was 7.5 mg of pentazocine hydrochloride or 17.5 mg of pethidine hydrochloride. Additional analgesia was provided at the discretion of the endoscopist when warranted by increased movement or expressions of suffering after starting the procedure.

Just after the endoscopic procedure, 0.5 mg of the benzodiazepine antagonist anexato was injected when the endoscopist determined that some sedative effect remained, and all patients rested in bed for at least 2 h after the procedure.

### 2.4. Statistical Analysis

Statistical analysis was performed using JMP Pro 16.0 software (SAS Institute, Cary, NC, USA). The correlations of individual demographic factors to pre-ESD D-dimer levels and to the change in D-dimer from before to after ESD were evaluated using Fisher’s exact test or Welch’s t-test. Factors correlating with pre-ESD D-dimer levels and the change in D-dimer from before to after ESD were analyzed using logistic regression analysis to control for the potentially confounding roles of age, thrombosis, hypertention, and antiplatelet drug use in the associations between pre-ESD D-dimer levels and demographic factors, as well as of age, ESD site, procedure duration, total or corrected midazolam dose, total pethidine hydrochloride dose, and total haloperidol dose in associations with the change in D-dimer from before to after ESD. Values of *p* < 0.05 were taken to indicate a significant difference.

The study plan was approved by the ethics committee of Teikyo University School of Medicine (approval no. 20-141).

## 3. Results

### 3.1. Patients

The study population comprised 117 patients. Patient demographics are shown in Table 1. The final pathological examination confirmed cancer in 90 patients. In patients with non-cancerous lesions, the diagnosis was gastric adenoma in 3 patients, colon adenoma in 17, serrated lesion of the colon in 4, and carcinoid tumor of the colon in 3. Pre-ESD D-dimer levels were within the normal range (<1.0 μg/mL) in 92 patients and above the upper limit of normal (≥1.0 μg/mL) in 25 patients. Nineteen patients used an antiplatelet drug (aspirin monotherapy, 14 patients; clopidogrel monotherapy, 2 patients; ticlopidine monotherapy, 1 patient; aspirin plus clopidogrel, 2 patients). One patient on aspirin monotherapy suspended aspirin use for more than 1 month before undergoing ESD, and one on clopidogrel monotherapy underwent ESD after suspending clopidogrel use for 7 days. The remaining patients stayed on aspirin or ticlopidine monotherapy through ESD. Ten patients used an anticoagulant. One patient on warfarin potassium remained on that treatment through ESD. Nine patients on a direct oral anticoagulant suspended use of that agent on the day of ESD.

### 3.2. Details of the ESD Procedure

Details of the ESD procedure are presented in Table 2. The median duration of ESD (duration of scope insertion) was 104 min (range, 70.5–154 min). Midazolam was used as the sedative for the procedure in 107 patients (91.5%), including 65 patients (98.5%) undergoing upper gastrointestinal ESD and 42 patients (82.4%) undergoing colonic ESD. Among midazolam users, the initial dose was 1.5 ± 0.6 mg and a sixfold difference was observed in initial midazolam doses given before the start of endoscopy in our study (0.5–3.0 mg). The mean total midazolam dose was 4.0 ± 2.7 mg in all patients, 5.4 ± 2.5 mg in those patients who underwent upper gastrointestinal ESD, and 1.8 ± 1.2 mg among those who underwent colonic ESD. All 29 patients who received additional haloperidol underwent upper gastrointestinal ESD (total dose, 4.8 ± 2.3 mg). Perforation occurred in 2 patients during colonic ESD. Each perforation was successfully treated with closure followed by fasting and antibiotics alone.

### 3.3. Relationships between Pre-ESD D-Dimer Level and Demographic Factors

The relationships between pre-ESD D-dimer level and demographic factors are presented in Table 3. Age, thrombosis, hypertension, and antiplatelet drug use were all significantly higher in the abnormal pre D-dimer level (≥1.0 μg/mL) group. In multivariate analysis, only age correlated significantly with abnormal D-dimer level (odds ratio (OR) = 1.1, 95% confidence interval (CI) 1.03–1.20; *p* = 0.009).

### 3.4. Relationships between Changes in D-Dimer Levels and Demographic Factors

This analysis comprised 109 patients, excluding 8 patients in whom pre-ESD D-dimer levels were >1.5 μg/mL. Mean D-dimer levels were 0.7 ± 0.3 μg/mL before ESD and 1.1 ± 0.9 μg/mL after ESD, with a mean change of 0.5 ± 0.9 μg/mL. Changes in D-dimer levels are presented for different demographic factors in Table 4. Age, ESD site (upper gastrointestinal ESD), procedure time, total midazolam dose, total pethidine hydrochloride dose, and total haloperidol dose all correlated with a change in D-dimer level ≥1.0 μg/mL. In multivariate analysis, total pethidine hydrochloride dose was the only factor that correlated significantly with a change in D-dimer level ≥1.0 μg/mL (OR = 1.0, 95%CI 0.94–0.99; *p* = 0.045).

### 3.5. Subgroup Analysis in Midazolam Users

To account for the heterogeneity in the sedative effect of midazolam among patients, we conducted a subgroup analysis in the 103 midazolam users using corrected midazolam doses (i.e., total midazolam dose divided by initial dose) (Table 5). Age, upper gastrointestinal ESD, procedure time, corrected midazolam dose, total pethidine hydrochloride dose, and total haloperidol dose correlated with a difference in D-dimer level ≥1.0 μg/mL. In multivariate analysis using these factors, only corrected midazolam dose correlated with post-ESD D-dimer elevation (OR = 1.5, 95%CI 0.43–0.95; *p* = 0.030).

## 4. Discussion

Sedation helps improve the results of diagnostic and therapeutic endoscopy and is a necessary part of therapeutic endoscopy [11]. Midazolam is an intravenous benzodiazepine sedative that shows less amnesic and phlebitic effects than other benzodiazepines, making this a widely used option in diagnostic and therapeutic endoscopy. In ESD and other time-consuming therapeutic endoscopic procedures, a benzodiazepine sedative is often combined with an analgesic to maintain moderate or greater sedation and allow safe, stable treatment. Our hospital primarily uses midazolam for sedation and the endoscopist’s choice of pentazocine hydrochloride or pethidine hydrochloride for analgesia.

We selected a threshold of 1.0 μg/mL for the change in D-dimer levels based on the normal range of D-dimer levels (<1.0 μg/mL) and a reported threshold of 1.9 μg/mL for postoperative D-dimer levels associated with post-ESD DVT [12]. We found that the corrected midazolam dose, as a value determined by dividing the total midazolam dose by the initial dose to correct for inter-individual differences in the sedative effect, correlated with increases in D-dimer levels following ESD of a gastrointestinal tumor. The sedative effect of midazolam shows marked inter-individual variability [17,18,19,20,21,22], as confirmed by the sixfold difference in initial midazolam doses given before the start of endoscopy in our study (0.5–3.0 mg). In early investigations, we suspected that the total midazolam dose might be involved in post-ESD D-dimer elevations, but multivariate analysis failed to confirm any such correlation. In searching for the reason, we realized that the large inter-individual differences in the sedative effect of midazolam could be involved and decided to conduct a subgroup analysis using midazolam doses corrected for these differences. Multivariate analysis revealed that the corrected midazolam dose did indeed correlate with post-ESD elevations in D-dimer levels.

The corrected midazolam dose indicates how many times greater the total dose was than the initial dose. The guidelines state that moderate or greater sedation is suitable for ESD [11]. Because the initial dose represents the dose required to achieve this level of sedation for a particular individual, the corrected midazolam dose should logically be proportional to the duration over which moderate or greater sedation is maintained. Our findings also indicate that post-ESD elevations in D-dimer correlated with the duration of sedation.

The total pethidine hydrochloride dose was shown to correlate with post-ESD elevations in D-dimer in the multivariate analysis using the total midazolam dose as one explanatory variable. Concomitant use of analgesia was known to enhance the sedative effect of midazolam; therefore, the correlation between the total pethidine hydrochloride dose and post-ESD elevations in D-dimer was thought to be reasonable. However, the total pethidine hydrochloride dose was not shown to correlate with this elevation in D-dimer in the subgroup analysis using the corrected midazolam dose. We are convinced that the corrected midazolam dose is more relevant in clinical practice than the total midazolam dose because the influence of sedation should be considered directly proportional to the effect of sedation. We thus consider that the results of our subgroup analysis are statistically relevant, and we concluded that the corrected midazolam dose was the only factor that correlated with post-ESD D-dimer elevation in this study. However, the correlation between total pethidine hydrochloride dose and post-ESD elevations in D-dimer could not be completely denied at the present stage; therefore, further research would be required to evaluate this correlation.

As a marker of elevated secondary fibrinolysis, D-dimer is elevated not only in DIC and VTE (including DVT), but also in inflammatory diseases, infections, cancer, hepatic cirrhosis, pregnant women, older individuals, cerebral infarction, and myocardial infarction [22]. Although our study examined D-dimer levels just before and after the therapeutic event of ESD, the elevation in D-dimer levels just after ESD should be due to either thrombi or inflammation, considering the mechanisms of D-dimer production. Although postoperative inflammation and infection are potential contributors to increased D-dimer levels, their effects appear not to have been involved because the white blood cell count (WBC) and C-reactive protein (CRP) the day after ESD did not correlate with increases in D-dimer, and those few patients who experienced intra-procedural perforation did not show elevated D-dimer levels. In addition, there are only a few patients with increases in D-dimer level ≥1.0 μg/mL in colonic ESD in which the tumor was resected in the same way as in upper GI ESD. Although these results would not directly show that inflammation resulting from tumor resection was unrelated to increases in D-dimer level, thrombi appear to have contributed greatly to the D-dimer elevations observed in our study. Previous reports of post-ESD DVT support this notion. This suggests that the duration of sedation, which correlated with elevated post-ESD D-dimer level, could pose a risk of DVT.

General anesthesia and surgery are not the only risk factors for DVT [23]. Abnormal coagulation appears to occur in patients with cancer when tumor cells activate the coagulation cascade, and vascular endothelial cells and macrophages promote coagulation and block anticoagulation [24]. Moreover, the presence of metastases and tumor volume was reported to correlate with abnormal coagulation in cancer [25,26]. Although our study focused on patients with gastrointestinal tumors, pre-procedural D-dimer levels of patients who had cancerous lesions did not differ significantly from those of patients who had non-cancerous lesions. The patients in our study primarily had early cancers localized to the mucosal layer, which are expected to lack metastases to lymph nodes. Abnormal coagulation is not expected to result from the small tumor volume in early cancer, which may explain why pre-procedural D-dimer levels did not differ between patients with and without cancerous lesions in our study. Moreover, because cancer status was not associated with post-ESD D-dimer elevations in the study, the early cancer targeted in ESD is unlikely to increase the risk of post-ESD VTE, including DVT.

Although concomitant use of analgesia would reduce the dose of sedative needed, methods for using analgesic agents such as pentazocine hydrochloride and pethidine hydrochloride varied widely between individual endoscopists in this retrospective study, so evaluating the effect of analgesia on the corrected midazolam dose was difficult in this retrospective study. Besides, as compared with the number of events, the number of explanatory variables was large in the logistic regression analysis used in this study. Therefore, the small sample size of this study also represents a limitation, and further investigations are required to verify the findings. In addition, we used midazolam as a sedative in our hospital. Although midazolam was usually used as a sedative for endoscopic treatment at a large number of endoscopic centers in Japan [11], other types of sedative such as propofol were also used for endoscopic treatment at many institutions around the world. Therefore, further investigations with regard to the correlation between other types of sedative and DVT will be needed in the future. Postoperative DVT prophylaxis, however, is recommended in the guidelines and standardized. Although ESD is not typically performed with general anesthesia, ESD for upper gastrointestinal tumors in particular requires sedation and, therefore, DVT prophylaxis such as compression stockings is ethically desirable for all types of ESD requiring extended sedation, regardless of whether cancer is involved.

## 5. Conclusions

This present study showed that the corrected midazolam dose correlated with increases in post-ESD D-dimer levels. This potential relationship indicates that patients undergoing ESD and requiring extended sedation may be at increased risk of DVT.

## Figures and Tables

**Table 1:** Demographic characteristics of patients.

	*N* = 117
Age (years)	70.7 (20–94)
Sex (male/female)	84 (71.8%)/33 (28.2%)
Organ (esophagus/stomach/colon)	17 (14.5%)/49 (41.9%)/51 (43.6%)
Pathological examination (cancerous/non-cancerous)	90 (76.9%)/27 (23.1%)
D-dimer (<1.0 μg/mL/≥1.0 μg/mL)	92 (78.6%)/25 (21.4%)
Comorbidities	
Thrombosis (absent/present)	88 (75.2%)/29 (24.8%)
Hypertension (absent/present)	51 (43.6%)/66 (56.4%)
Hyperlipidemia (absent/present)	82 (70.1%)/35 (29.9%)
Diabetes mellitus (absent/present)	90 (76.9%)/27 (23.1%)
Dialysis (absent/present)	114 (97.4%)/3 (2.6%)
Antiplatelet drug use (absent/present)	98 (83.8%)/19 (16.2%)
Anticoagulant use (absent/present)	107 (91.5%)/10 (8.5%)

**Table 2:** ESD procedure details.

	*N* = 117
Duration (min)	104 (70.5–154)
Maximum diameter of resected sample (mm)	31.9 ± 14.4
Midazolam users (*n*)	107/117 (upper GI ESD: 65/66; colonic ESD: 42/51)
Total midazolam dose (mg)	4.0 ± 2.7
Total dose in upper GI ESD (mg)	5.4 ± 2.5
Total dose in colonic ESD (mg)	1.8 ± 1.2
Initial midazolam dose (mg)	1.5 ± 0.6
Pentazocine hydrochloride users (*n*)	51/117 (upper GI ESD: 18/66; colonic ESD: 33/51)
Total pentazocine hydrochloride dose (mg)	15.0 ± 4.3
Pethidine hydrochloride users (*n*)	61/117 (upper GI ESD: 45/66; colonic ESD: 16/51)
Pethidine hydrochloride dose (mg)	45.3 ± 18.7
Patients with haloperidol added (*n*)	29/117 (upper GI ESD: 29/66; colonic ESD: 0/51)
Total haloperidol dose added (mg)	4.8 ± 2.3
Complications (perforation) (*n*)	2/117 (upper GI ESD: 0/66; colonic ESD: 2/51)
WBC the day before ESD (10^9^/L)	6.13 ± 2.09
WBC the day after ESD (10^9^/L)	7.63 ± 2.42
CRP the day before ESD (mg/dL)	0.11 ± 0.16
CRP the day after ESD (mg/dL)	0.72 ± 1.04

Continuous value variables are described mean ± standard deviation. WBC, white blood cell count; CRP, C-reactive protein.

**Table 3:** Associations between pre-ESD D-dimer levels and demographic factors.

	Pre-ESD D-Dimer Level					
	<1.0 (*n* = 92)	≥1.0 (*n* = 25)	Univariate	OR	95%CI	Multivariate
Sex (male/female)	68/24	16/9	0.328			
Age (years)	69.0 ± 1.2	77.1 ± 2.2	<0.001 ^a^	1.1	1.03–1.20	0.009 ^a^
Pathological examination (cancerous/non-cancerous)	73/19	17/8	0.285			
Thrombosis (*n*)	18	11	0.018 ^a^	1.2	0.27–4.66	0.804
Hypertension (*n*)	46	20	0.006 ^a^	2.3	0.76–7.95	0.158
Hyperlipidemia (*n*)	24	11	0.091			
Diabetes mellitus (*n*)	19	8	0.285			
Dialysis (*n*)	2	1	0.517			
Antiplatelet drug use (*n*)	11	8	0.029 ^a^	2.5	0.56–12.69	0.234
Anticoagulant use (*n*)	7	3	0.443			

Continuous value variables are described as mean ± standard deviation. ^a^: *p* < 0.05.

**Table 4:** Associations with a change in D-dimer from before to after ESD.

	Change in D-Dimer from before to after ESD					
	<1.0 (*n* = 89)	≥1.0 (*n* = 20)	Univariate	OR	95%CI	Multivariate
Sex (male/female)	61/28	17/3	0.177			
Age (years)	68.9 ± 11.84	74.4 ± 7.23	0.034 ^a^	1.1	0.86–1.00	0.074
Pathological examination (cancerous/non-cancerous)	67/22	18/2	0.233			
ESD site (upper/lower)	47/42	17/3	0.011 ^a^	2.1	0.38–13.64	0.419
Maximum diameter of resected sample (mm)	31 ± 14.4	36 ± 14.4	0.104			
WBC the day after ESD (10^9^/L)	8.0 ± 2.4	7.3 ± 2.5	0.364			
CRP the day after ESD (mg/dL)	0.6 ± 0.9	1.2 ± 1.5	0.068			
Thrombosis (*n*)	24	3	0.391			
Hypertension (*n*)	48	11	1.000			
Hyperlipidemia (*n*)	27	4	0.423			
Diabetes mellitus (*n*)	19	7	0.246			
Dialysis (*n*)	1	1	0.335			
Antiplatelet drug use (*n*)	14	3	1.000			
Anticoagulant use (*n*)	9	0	0.206			
Procedure duration (min)	113.7 ± 63.2	147.6 ± 65.9	0.028 ^a^	1.0	0.99–1.00	0.202
Total midazolam dose (mg)	3.5 ± 2.8	5.5 ± 2.4	<0.001 ^a^	1.0	0.73–1.27	0.757
Total pentazocine Hydrochloride dose (mg)	7.8 ± 8.1	1.1 ± 5.0	<0.001 ^a^			
Total pethidine Hydrochloride dose (mg)	20.1 ± 24.9	45.5 ± 25.0	<0.001 ^a^	1.0	0.94–0.99	0.045 ^a^
Total haloperidol dose (mg)	1.0 ± 2.2	2.6 ± 3.3	0.011 ^a^	1.0	0.79–1.26	0.962

Continuous value variables are described as mean ± standard deviation. WBC, white blood cell count; CRP, C-reactive protein. ^a^: *p* < 0.05.

**Table 5:** Associations with a change in D-dimer from before to after ESD in the subgroup analysis of midazolam users.

	Change in D-Dimer from before to after ESD					
	<1.0 (*n* = 83)	≥1.0 (*n* = 20)	Univariate	OR	95%CI	Multivariate
Sex (male/female)	57/26	17/3	0.177			
Age (years)	68.7 ± 12.07	74.4 ± 7.23	0.034 ^a^	1.1	0.87–1.00	0.098
Pathological examination (cancerous/non-cancerous)	64/19	18/2	0.233			
ESD site (upper/lower)	47/36	17/3	0.011 ^a^	1.2	0.25–6.71	0.818
Maximum diameter of resected sample (mm)	31 ± 14.8	36 ± 14.4	0.104			
WBC the day after ESD (10^9^/L)	7.9 ± 2.4	7.4 ± 2.5	0.364			
CRP the day after ESD (mg/dL)	0.6 ± 0.9	1.2 ±1.5	0.068			
Thrombosis (*n*)	18	3	0.391			
Hypertension (*n*)	43	11	1.000			
Hyperlipidemia (*n*)	23	4	0.423			
Diabetes mellitus (*n*)	17	7	0.246			
Dialysis (*n*)	1	1	0.335			
Antiplatelet drug use (*n*)	9	3	1.000			
Anticoagulant use (*n*)	8	0	0.206			
Procedure duration (min)	113.3 ± 65.0	147.6 ± 65.9	0.028 ^a^	1.0	0.99–1.00	0.202
Corrected midazolam doses	2.3 ± 1.3	3.9 ± 1.8	<0.001 ^a^	1.5	0.43–0.95	0.030 ^a^
Total pentazocine Hydrochloride dose (mg)	7.9 ± 8.2	1.1 ± 5.0	<0.001 ^a^			
Total pethidine Hydrochloride dose (mg)	20.5 ± 25.5	45.5 ± 25.0	<0.001 ^a^	1.0	0.95–1.00	0.076
Total haloperidol dose (mg)	1.1 ± 2.2	2.6 ± 3.3	0.011 ^a^	1.0	0.77–1.28	0.959

Continuous value variables are described as mean ± standard deviation. WBC, white blood cell count; ESD, Endoscopic submucosal dissection; CRP, C-reactive protein. ^a^: *p* < 0.05.

## Data Availability

The data supporting the findings of this study are available on request from the corresponding author, Shinya Kodashima (kodashima-tky@umin.ac.jp) at Teikyo University School of Medicine. The data are not publicly available due to the inclusion of information that could compromise the privacy of research participants.
